# Polar Vortex Multi-Day Intensity Prediction Relying on New Deep Learning Model: A Combined Convolution Neural Network with Long Short-Term Memory Based on Gaussian Smoothing Method

**DOI:** 10.3390/e23101314

**Published:** 2021-10-08

**Authors:** Kecheng Peng, Xiaoqun Cao, Bainian Liu, Yanan Guo, Chaohao Xiao, Wenlong Tian

**Affiliations:** 1 College of Meteorology and Oceanography, National University of Defense Technology, Changsha 410000, China; pengkc@nudt.edu.cn (K.P.); bnliu@nudt.edu.cn (B.L.); xiaochaohao20@nudt.edu.cn (C.X.); tianwl@nudt.edu.cn (W.T.); 2 College of Computer, National University of Defense Technology, Changsha 410000, China; 3 Trainer Simulation Training Center, Naval Aeronautical University, Huludao 125000, China; guoyn14@lzu.edu.cn

**Keywords:** polar vortex intensity, Gaussian smoothing, three-dimensional convolutional neural network, long short-term memory network

## Abstract

The variation of polar vortex intensity is a significant factor affecting the atmospheric conditions and weather in the Northern Hemisphere (NH) and even the world. However, previous studies on the prediction of polar vortex intensity are insufficient. This paper establishes a deep learning (DL) model for multi-day and long-time intensity prediction of the polar vortex. Focusing on the winter period with the strongest polar vortex intensity, geopotential height (GPH) data of NCEP from 1948 to 2020 at 50 hPa are used to construct the dataset of polar vortex anomaly distribution images and polar vortex intensity time series. Then, we propose a new convolution neural network with long short-term memory based on Gaussian smoothing (GSCNN-LSTM) model which can not only accurately predict the variation characteristics of polar vortex intensity from day to day, but also can produce a skillful forecast for lead times of up to 20 days. Moreover, the innovative GSCNN-LSTM model has better stability and skillful correlation prediction than the traditional and some advanced spatiotemporal sequence prediction models. The accuracy of the model suggests important implications that DL methods have good applicability in forecasting the nonlinear system and vortex spatial–temporal characteristics variation in the atmosphere.

## 1. Introduction

### 1.1. Concept and Research Background

The Arctic polar vortex, hereinafter referred to as polar vortex, is one of the major atmospheric circulation systems affecting the atmospheric conditions and weather in the Northern Hemisphere (NH). It plays a critical role in feedback mechanism of stratosphere–troposphere exchange and high-latitude weather circulation system. The formation of the polar vortex directly affects the polar stratosphere–troposphere exchange (STE) process and polar environment, and the variation of its intensity and position inevitably leads to the anomaly of circulation, which is specifically reflected in its impact on temperature and precipitation [1,2,3,4]. The location and intensity of the polar vortex are closely related to the cold air activities in the Eurasian continent [1,5,6,7], which also have strong interaction with El Niño–Southern Oscillation (ENSO) [8,9,10], North Atlantic Oscillation (NAO) [11,12], Quasi-biennial Oscillation (QBO) [13,14,15], and other atmospheric circulation systems. Besides, from the perspective of Earth ecology and environment, the polar vortex also plays a significant role in Arctic sea ice loss [16,17] and global warming feedback mechanism [18,19].

The intensity variation of the polar vortex is a complicated nonlinear system. In recent years, many previous studies have pointed out that the intensity drastic variation and fragmentation process of the polar vortex will lead to more extreme weather events, such as cold waves and strong snowfall [5,6,7]. Strengthening of polar vortex intensity will lead to low ozone values, which may have a connection with air pollution and extreme weather events [20,21], further causing sudden changes in the global ecological environment and affecting human health. Among the studies on the effect of polar vortex intensity, Baldwin et al. [22] pointed out that the breaking and weakening of the polar vortex are accompanied by the rise of stratospheric temperature and potential height, which affect the global weather change on the surface, and the weak polar vortex event also can cause temperature fluctuations in the lower troposphere and cause the Arctic Oscillation (AO) to have a continuous negative phase [12]. Oehrlein et al. [23] found that the strong polar vortex events and variability have a certain degree of response under chemical action (mainly ozone), indicating that interaction between polar vortex intensity and chemical substances is an important system that can represent the climate change in winter in the North Atlantic and Europe. The impact of this variation of polar vortex intensity on many extreme weather events and the role of chemicals show that it is of great significance to accurately predict the polar vortex intensity.

Current studies have illustrated that there exists an obvious interannual and decadal variation in polar vortex intensity [19,24,25,26], while there are few previous types of research focused on the variation of daily scale intensity, and most of them focus on the case study of strong/weak polar vortex intensity events in seasons and impact on extreme weather events and the role of chemical substances [12,23,27]. It shows a strong uncertainty characteristic in the prediction of almost all nonlinear indexes in the atmosphere, and the model finds it difficult to capture the physical rules of the nonlinear system itself, such as ENSO index, tropical cyclone (TC) intensity, and the prediction of extratropical cyclone activity, e.g., [28,29,30,31,32,33]. Therefore, the study of the prediction of polar vortex intensity on the day-to-day scale is a new direction that is worth exploring. It can further predict the impact of the polar vortex on atmospheric circulation systems during the intraseasonal and improve the probability of predicting extreme events.

In the field of atmospheric and ocean science, plenty of atmospheric circulation phenomena and the prediction of various weather system indexes provide an important basis for the prediction of polar vortex intensity. For example, Gray et al. [34] utilized the positive prediction model to predict polar vortex events, demonstrating that better reflecting the characteristics of vortex flow above the stratosphere can increase the predictability of the polar vortex. Zheng et al. [29] used NMME and S2S models to predict the winter extratropical cyclone activities from sub-seasonal scale to seasonal scale, pointing out that the sub-seasonal prediction technology in East Asia and other regions is related to the anomaly of the stratospheric polar vortex. Lee et al. [35] compared six prediction systems to predict stratospheric polar vortex and positive tropospheric Arctic Oscillation (AO) in winter of the NH. The results show that there exists a strong correlation between the accuracy of the stratospheric vortex and AO prediction. These studies demonstrated the importance of polar vortex intensity prediction, but they did not clearly predict the day-to-day variation of the polar vortex, and the polar vortex events are always predicted by the numerical prediction model. Thus, accurately predicting the variation of polar vortex intensity and capturing the characteristics of polar vortex distribution can not only provide important research guidance for the prediction of various nonlinear systems affecting the global atmosphere and climate change, but also optimize the advanced methods for various vortex intensity indexes forecasting.

According to the present research, the main methods used in predicting the nonlinear exponential variation in the atmosphere are traditional mathematical methods and model prediction, while traditional mathematical–statistical methods have many defects. It can only learn the sequence characteristics of the data, while it cannot capture the physical rules of the atmosphere. Traditional mathematical–statistical methods such as autoregression (AR), moving average (MA), and autoregression integrated moving average (ARIMA) cannot capture the spatial distribution information of physical phenomena in the atmosphere when predicting time series [36,37,38,39,40,41]. Similar to TCs and many synoptic-scale vortices, the distribution modality of the polar vortex will change greatly in a few days or even a few hours. The role of physical variables and dynamic fields should be fully considered. Moreover, the polar vortex intensity index varies rapidly on the daily time scale, and traditional models cannot capture the signal of intensity variation very well, so it is necessary to further analyze the temporal and spatial characteristics.

### 1.2. Related Works and Research Gap

Deep learning (DL) is an important branch of artificial intelligence (AI). With the advent of the information age, DL methods play an important role in various fields of natural science research and have made outstanding contributions. Nosratabadi et al. [42] pointed out that the algorithm of hybrid DL models will be further applied to various fields of data science, including the stock market, marketing, and cryptocurrency. With the extensive development of computer science and statistics, AI also promotes the application of DL in Earth science, hydrological processes, and climate change, giving the DL algorithm natural applicability in the application field of weather forecasting [43]. DL algorithms and models have the ability to learn from a large number of long-time signal data and extract image features, that is, providing a powerful nonlinear function fitting ability to forecast the polar vortex index. Compared with ordinary atmospheric models and traditional mathematical methods, many DL neural networks have strong advantages [44,45,46,47,48].

In recent years, traditional DL models such as multilayer perceptron (MLP), recurrent neural network (RNN) and convolutional neural network (CNN) for learning multidimensional fields have been widely used in the atmosphere and ocean [49,50,51,52,53,54,55]. When using traditional DL methods for time series prediction and image feature extraction, more accurate prediction results can usually be obtained by adjusting the parameters and structure of the network model. For example, Ham et al. [30] have shown that, compared with many atmospheric models, CNN will achieve a higher correlation when predicting the El Niño index and lead ENSO events forecast time by one and a half years. Deng et al. [56] further proposed a vortex identification method, based on CNN, which can quickly detect vortices from the flow field in an objective and robust way, and addressed the defects of traditional methods. Similar to the vortex structure of polar vortex, there is a lot of research on TC track and intensity prediction, e.g., [31,57,58,59,60,61]. For example, the MLP network was used to predict the position of the cyclone eye in a high-resolution 3D remote sensing image by [62]. Alemany et al. [63] considered all types of hurricanes, then used RNN to predict hurricane trajectory by adding the grid identification number to learn the spatial relationship on the map. A better prediction accuracy than the results of [64] was obtained. Rüttgers et al. [31] used typhoon satellite images as the input to a generative adversarial network (GAN). After adding dynamic fields such as velocity field, the prediction of typhoon trajectory was significantly improved. The long short-term memory (LSTM) network was first proposed by [65], and many of its variant structures and applications were derived. Sutskever et al. [66] provided a general framework for sequence-to-sequence learning by applying LSTM encoder–decoder framework. Karevan et al. [55] investigated many achieved remarkable results in the weather prediction tasks through LSTM and its improved models.

Numerous previous studies indicate that CNN, LSTM, and their improved models are also widely used in different scientific research fields. Similar to the description of [67], we further list some of the latest representative applications of CNN and LSTM in various scientific fields, as shown in Table 1:

However, due to the chaotic characteristics and high uncertainty of various weather systems in the atmosphere, many extreme weather events and meteorological ocean indexes show complex variation characteristics. The ordinary machine learning model cannot extract the change characteristics of these systems well, and the prediction accuracy also reaches a bottleneck. For example, the variation of polar vortex intensity and location are closely related to sea surface temperature (SST) and sea ice loss in the Arctic, but the characteristics of such uncertain influence factors and physical information need to be further extracted by complex networks. The DL model is also developing in the direction of deeper and wider.

With the renewal and iteration of the neural networks, many spatiotemporal prediction models are constantly proposed and applied to the prediction of various systems in the atmosphere [76,77,78,79,80,81,82,83]. The ensemble DL model is one of the most typical cases. Compared with the traditional DL models, ensemble models often achieve better prediction results, which can capture the important features in images and time sequences more accurately. A mask region-based convolutional neural network (mask R-CNN) model for quasi-supervised reidentification of tropical cyclones proposed by [84] showed a good performance in the field of cyclone identification. Lguensat et al. [76] introduced Eddynet, which can automatically detect and classify eddy currents from sea surface height (SSH) maps, providing a simple and powerful tool for the marine remote sensing community. In the research of weather phenomenon prediction, a data-driven model based on a neural network, called Lightnet, for lightning prediction was proposed by [77]. The experimental results illustrated that Lightnet can achieve a threefold improvement in equitable thread score for six hours prediction compared with the other three models. The convolutional LSTM (ConvLSTM) network was first proposed by [85] for short-term precipitation prediction, which produced better prediction results than the traditional model. Moreover, ConvLSTM has been widely improved and applied to many fields, such as feature recognition and spatiotemporal prediction [82,86,87]. Similarly, the application of SmaAt-UNet proposed by [78] to short-term precipitation can also make up for the defection of numerical weather forecast to use the latest information for a short-term forecast. The generation of streamline model is also an essential way to analyze the meteorological; Lee et al. [81] described the flow field based on the three-dimensional U-net regression model and line integral convolution (LIC) volume with remarkable speed and visualization effect. With the development of mathematical and physical, many mathematical methods for time series and image processing have been gradually proposed and updated.

In order to better approximate the real value and improve the training efficiency of the DL models, some of the latest studies suggest that plenty of advanced methods also can be improved for completing the tasks with higher efficiency. For example, the Big Bird, which is a spark attention mechanism proposed by [88] can greatly improve the performance of various NLP tasks such as answering questions and summarizing. This study also proposes new applications of genomic data. In the research of [89], which is based on the most advanced time series model called transformer, the mathematical improved method was applied to express the self-attention in the transformer as the linear point product of the kernel feature map, and the combination of the matrix product was used to reduce the complexity. The improved method has a speed of up to 4000 times in the autoregressive prediction of very long sequences. Previous studies also have shown that adding mathematical processing methods to the machine learning model will improve the stability and fitting effect of the model to a certain extent. For example, Peng et al. [79] investigated that the CEEMDAN + ConvGRU method can accurately predict the intensity of the South Asian high (SAH) and achieved better stability than the traditional machine learning method. The ensemble empirical mode decomposition (EEMD) combined with CNN + LSTM method proposed by [90] also can predict the El Niño index more accurately and stably.

### 1.3. Research Significance and Contribution

As per the significant research we mentioned above, the development of DL models has made numerous achievements in atmospheric prediction. Thus, it is feasible to combine the ensemble DL model with advanced mathematical methods to extract the distribution characteristics of the polar vortex for intensity prediction.

To the best of our knowledge, there is no research on using the DL method to extract polar vortex image features and predict polar vortex intensity index on an intraseasonal scale. The prediction accuracy of polar vortex intensity index is determined by the abnormal distribution of potential height. Therefore, it is feasible to use CNN and LSTM methods in DL combined with the latest signal processing and data smoothing methods to predict the variation of polar vortex intensity index. Due to that the traditional two-dimensional CNN model cannot meet the prediction of multiple time steps, in order to further capture the spatial characteristics of polar vortex distribution, a three-dimensional convolution neural network (3DCNN) is used to extract the characteristics of polar vortex images after two-dimensional Gaussian smoothing. After completing the convolution process, it is necessary to capture the time series characteristics of polar vortex intensity, and the prediction results of multiday polar vortex intensity index are obtained through training the LSTM network with one-dimensional Gaussian smoothing time series data as input. Furthermore, traditional and advanced DL models are also used for comparison.

The accurate prediction of polar vortex intensity can bring important scientific significance to the field of meteorological research. Firstly, it can provide a reference basis for the loss of sea ice and the occurrence of extreme weather phenomena in the Arctic region. After the polar vortex intensity predicted by the DL model is added to the model system as a prediction factor, the numerical prediction model can more accurately predict the weather conditions in the areas affected by vortex. In terms of intraseasonal scale, more accurate prediction of multiday polar vortex intensity index will provide reference for the establishment and optimization of the prediction model of nonlinear system in the atmosphere. In addition, the high robustness and accuracy of machine learning (ML) and DL methods provide a basis for polar vortex prediction in the field of geoscience research [43]. At present, there exist many uncertainties in the research of chemical and physical processes under atmosphere environment by using the variation of polar vortex intensity; the function of weather forecast is to better forecast weather phenomena after finding out the correlation of various systems. Therefore, this study can provide a more accurate basis for the interaction between the polar vortex and various systems in the atmosphere and add further improved sea and air interaction and land and air interaction factors to the model, so as to provide a reference for the in-depth study of the impact of the atmospheric vortex system on weather prediction. The positive and negative phase prediction of AO and ENSO events can also further improve the accuracy under the intraseasonal prediction of polar vortex intensity [8,12].

However, the existing research and dataset did not elaborate on the prediction of many vortex systems in the atmosphere. In order to solve the problem in a proper way, this paper further explores whether the application of the DL model can better solve the prediction problems of nonlinear systems in the atmosphere, such as the variation of polar vortex intensity. How to find an appropriate model for comparative analysis is also one of the key research problems of this paper, according to the spatiotemporal sequence characteristics of polar vortex intensity, whether a new model structure or improved method needs to be proposed by comparing the traditional and advanced DL image prediction and time series prediction models. Since the formation and development of the weather system are affected by many meteorological elements, which can be regarded as prediction factors in DL models, how to add appropriate variables and deal with the relationship between image and time series are the key and difficult points in the prediction process. Can the temperature field, potential vorticity field, and flow field be added to the time sequence information to obtain higher prediction accuracy?

### 1.4. Organization of the Paper

In this study, we propose a new DL model for predicting time series using image information. It mainly focuses on the polar vortex feature extraction process in the constructed polar vortex distribution image information database, followed by the input timing and image smoothing process. The polar vortex intensity time series and image database are constructed from the geopotential height (GPH) data provided by the National Centers for Environmental Prediction and the National Center for Atmospheric Research (NCEP/NCAR). In order to predict the temporal characteristics of the polar vortex more accurately, we remove noise in the data with Gaussian smoothing techniques. The reconstructed data is input into the 3DCNN-LSTM model to obtain the results. The predicted multiday polar vortex intensity index is compared with the real polar vortex index to obtain the correlation and compared with the traditional neural network model to test the stability of the method and evaluate the ability of the method to predict the nonlinear system in the atmosphere.

The main structure of this paper is as follows: firstly, the construction of polar vortex image and polar vortex intensity dataset, Gaussian smoothing method, traditional neural network method, and the proposed innovative neural network model convolution neural network with long short-term memory based on Gaussian smoothing (GSCNN-LSTM) are introduced in the second part; then, the prediction accuracy of various network models is investigated and the stability of the model is evaluated in the third part; the final part summarizes the conclusions of the article and looks forward to the future research direction.

## 2. Model and Methods

### 2.1. Dataset Construction

Because the polar vortex is one of the most powerful vortex and weather systems in the NH, its intensity variation can be explained by the variable field of many meteorological elements. In this study, the most standard and widely used polar vortex intensity definition method is adopted, that is, the dynamic field of the polar vortex is expressed by the abnormal variation of geopotential height (GPH) field. Previous studies have shown that the variation of the GPH field has a strong correlation with the dynamic structure of various vortex systems and the distribution of meteorological element field, and the modes and structures of a medium, small-scale, and synoptic-scale vortices can be characterized and studied by GPH, e.g., [12,91,92]. Therefore, in order to construct a set database with a real and sufficient intensity index, we selected the GPH data from the National Centers for Environmental Prediction and National Center for Atmospheric Research (NCEP/NCAR) from January to March and December (DJFM) in 1948–2020 and took the daily average data as the dataset for calculating the polar vortex intensity index. Since the polar vortex has the strongest intensity at 50–10 hPa, we use the GPH field at the height of the strongest polar vortex in winter, that is, the 50 hPa isobaric surface as the horizontal field calculated by the intensity index. The polar vortex intensity is defined by the abnormal variation of GPH combined with the variation of latitudes, as follows:

(1) Za≡∑Z/8852

(2) $$Z^{\prime }\equiv Z-Za$$

(3) $$-Zp\equiv -\sum (Z^{\prime }\cos\varphi )/\sum \cos\varphi$$

The anomaly of the polar weighted average GPH is used to represent the intensity index of the polar vortex, where Z and Za in Equations (1) and (2) represent the daily averaged GPH and all selected days (8852 in total) averaged GPH, respectively, $Z^{\prime }$ in Equations (2) and (3) represents the anomaly of GPH after removing the effect of year cyclic, and φ indicates the latitude variation. In the process of calculating the intensity index, the abnormal value $Z^{\prime }$ of the potential height needs to be calculated first, and the polar vortex intensity index −Zp is opposite to the sign of the GPH anomaly in the polar region, so the positive polar vortex intensity index corresponds to the strong polar vortex, and the negative intensity index indicates the weak polar vortex.

The database construction process of polar vortex images and intensity index series is as follows: Primarily, because the selected months (DJFM) of the polar vortex in each year are not all continuous, different from the traditional vortex variation, this study needs to splice the December of the previous year and January to March of the next year to form the variation of the polar vortex in this year. Secondly, because there is a difference between a leap year and normal year in February of each year, the number of days selected in a normal year is 121 days, and that in a leap year is 122 days. Based on the existing NCEP/NCAR database, we first constructed 122 characteristic maps of polar potential height anomaly distribution multiplied by 19, adding 121 characteristic maps multiplied by 54 (122 × 19 + 121 × 54), a total of 8852, then the daily polar vortex intensity index is calculated through Equation (1), and the time series database of polar vortex intensity index with a total length of 8852 is also constructed in the same way.

### 2.2. Gaussian Smoothing (GS)

Gaussian smoothing (GS) is also called Gaussian blur. This method is similar to convolution. It is widely used in the research of image blur, image classification, and detection. GS can effectively remove the details and noise of the image. In this sense, it is similar to the mean filter, but uses different kernels to represent the Gaussian hump shape (bell shape). The Gaussian kernel used in this study is mainly two-dimensional Gaussian kernel and one-dimensional Gaussian kernel, which are used for the denoising of polar vortex intensity index sequence and the smoothing of polar vortex distribution images, respectively.

(4) $$G\left(x\right)=\frac{1}{\sqrt{2\pi }\sigma }e^{-\frac{x^{2}}{2\sigma^{2}}}$$

(5) $$G\left(x,y\right)=\frac{1}{2\pi \sigma^{2}}e^{-\frac{x^{2}+y^{2}}{2\sigma^{2}}}$$

(6) $$\frac{1}{273}\left[\begin{array}{ccccc} 1 & 4 & 7 & 4 & 1\\ 4 & 16 & 26 & 16 & 4\\ 7 & 26 & 41 & 26 & 7\\ 4 & 16 & 26 & 16 & 4\\ 1 & 4 & 7 & 4 & 1 \end{array}\right]$$

σ in Equations (4) and (5) is the standard deviation of normal distribution, and its value determines the decay rate of the function. x represents the variable value, which refers to the polar vortex intensity index. The matrix of Equation (6) shows a suitable integer valued convolution kernel, which is similar to the Gaussian distribution at σ=1.0. Due to this, the Gaussian filter determines the weight by spatial distance, while it cannot consider using color distance to determine the weight. As a result, Gaussian filtering not only removes noise, but also blurs the boundary to a certain extent. Therefore, in order to further reduce the error of polar vortex distribution in the process of GS, we first smooth the global potential height anomaly distribution at 50 hPa, and then eliminate the edge effect. At last, the characteristic map of the polar vortex in the polar region is intercepted and included in the image database.

### 2.3. Three-Dimensional Convolutional Neural Network (3DCNN), Long Short-Term Memory (LSTM), Convolutional LSTM (ConvLSTM)

In the field of DL, there are plenty of networks for image learning, including image recognition and image classification, among which the most traditional method is convolutional neural network (CNN). The three-dimensional convolutional neural network (3DCNN) mentioned in this study is composed of convolution layer, average pooling layer, and full connection (FC) layer. The CNN uses multiple convolution filters in the convolution process. Generally, the input channel (depth) of the output shape after convolution is the number of filters. We generally refer to filter as the number of convolution kernels. When extracting image features, the two-dimensional convolution neural network (2DCNN) only performs convolution operation for the image of a single time step. Because this recognition does not take the information of time dimension into account, in order to accurately predict the polar vorticity of multiple time steps (multiple days) in this study, it is necessary for the forecast lead multiday time to use the polar vortex distribution feature information of the previous few days, or even tens of days. According to the requests, a multiday input network will take advantage of 3DCNN functions. This means the time information dimension is added to the two-dimensional image information as the input of the convolution layer, and then the three-dimensional data are convoluted, and local features are extracted to further obtain the feature matrix. The pooling layer mainly downsamples the feature matrix and plays a secondary role to extract the features of images. The pooling operation can reduce the dimension and prevent overfitting. Note that the average pooling layer is used for feature extraction in this study. With the increase of the convolution layer, the image features extracted by 3DCNN will become more and more abstract. After the FC, the prediction results are obtained by outputting the layer activation function relu.

Long short-term memory (LSTM) is a model extended from recurrent neural network (RNN), which can also be called fully connected LSTM (FC-LSTM). Traditional RNN has defects in dealing with long-term memory, which is prone to gradient explosion or gradient disappearance. On the basis of RNN, LSTM adds the concept of three gates of memory cells, namely input gate, output gate, and forget gate. Through these three gates, control information is added to or forgotten from the memory unit, so that it can provide clear long-term memory and learn long-term rules. Thus, the problem of gradient explosion or gradient disappearance caused by the increase of RNN data volume is solved. The calculation process of LSTM is mainly as follows:

(7) $$Input\;Gate:i_{t}=\sigma \left(W_{i}\cdot \left[h_{t-1},x_{t}\right]+b_{i}\right)$$

(8) $${\tilde{C}}_{t}=tanh\left(W_{c}\cdot \left[h_{t-1},x_{t}\right]+b_{f}\right)$$

(9) $$Cell\;State:C_{t}=f_{t}*C_{t-1}+i_{t}*{\tilde{C}}_{t}$$

(10) $$Gorget\;Gate:f_{t}=\sigma \left(W_{f}\cdot \left[h_{t-1},x_{t}\right]+b_{f}\right)$$

(11) $$Output\;Gate:o_{t}=\sigma \left(W_{o}\cdot \left[h_{t-1},x_{t}\right]+b_{o}\right)$$

(12) $$h_{t}=o_{t}*tanh\left(C_{t}\right)$$

$f_{t}$ represents the forget gate; $i_{t}$ represents the input gate; o is the output gate; ${\tilde{C}}_{t}$ indicates the unit status of the current input, and $h_{t}$ is the final output. “·” represents the element-wise multiplication operation; σ represents the sigmoid activation function; “∗” represents the convolution calculation, also known as Hadamard product. It represents the multiplication operation of two matrices with the same dimension. The sigmoid layer outputs a number between 0 and 1 to describe how much each information vector should pass. A value of zero means “do not pass any information”, while a value of 1 means “let all information pass”. The calculation process of the LSTM network is shown in Equation (7). When the two inputs $x_{t}$ and $h_{t-1}$ enter the LSTM cell together, the forget gate $f_{t}$ will determine which information in the old state should be forgotten according to the learned weight $W_{f}$. In Equation (9), by calculating $f_{t}$ and the state ${\tilde{C}}_{t}$ of the previous cell, the Hadamard product can filter out unimportant information. Next, the input gate calculates the updated information and creates a new cell state $C_{t}$. As shown in Equations (7) and (8), the information to be updated can be obtained by multiplying the two, and the current state can be updated by adding the filtered information to the information to be updated. Finally, as shown in Equations (11) and (12), the output gate $o_{t}$ will obtain the input value through the sigmoid function and obtain the state $C_{t}$ just calculated by the cell with the tanh function, $o_{t}$*,* multiplied by $C_{t}$ to obtain the output of this operation.

The classical LSTM structure expands the data into one-dimensional for prediction, which can better solve the time correlation, while FC-LSTM can only extract the time series information and cannot extract the spatial information. Spatial data, especially radar echo data, contains a lot of redundant information, which implies that FC-LSTM cannot appropriately process the datasets. Therefore, convolutional LSTM (ConvLSTM) with convolution structure between the input-to-state and state-to-state of LSTM is proposed by [85]. The structure of ConvLSTM is similar to that of LSTM, except that ConvLSTM introduces convolution operation, and its calculation process is shown in Equations (13) and (17):

(13) $$i_{t}=\sigma \left(W_{xi}*X_{t}+W_{hi}*H_{t-1}+W_{ci}\circ C_{t-1}+b_{i}\right)$$

(14) $$f_{t}=\sigma \left(W_{xf}*X_{t}+W_{hf}*H_{t-1}+W_{cf}\circ C_{t-1}+b_{f}\right)$$

(15) $$C_{t}=f_{t}\circ C_{t-1}+i_{t}\circ tanh\left(W_{xc}*X_{t}+W_{hc}*H_{t-1}+b_{c}\right)$$

(16) $$o_{t}=\sigma \left(W_{xo}*X_{t}+W_{ho}*H_{t-1}+W_{co}\circ C_{t}+b_{o}\right)$$

(17) $$H_{t}=o_{t}\circ tanh\left(C_{t}\right)$$

where “∗” represents the convolution calculation, the weight W is a two-dimensional convolution kernel, and the three gates of cell state $C_{t}$, hidden state $H_{t}$, and $i_{t}$, $f_{t}$, and $o_{t}$ are all three-dimensional tensors. However, compared with ConvLSTM’s ability to extract and accurately predict information in spatiotemporal sequence, this study tests and verifies the effect of the model in multi-time step prediction and multi-time step input; the result shows that in the appropriate new network of 3DCNN combined with LSTM, the prediction result is more accurate than ConvLSTM. Therefore, by adjusting the network structure and number of layers of 3DCNN, combined with LSTM, and introducing GS algorithm into the model, more accurate and stable results can be obtained.

### 2.4. Innovative Training Methods

**Figure 1:**
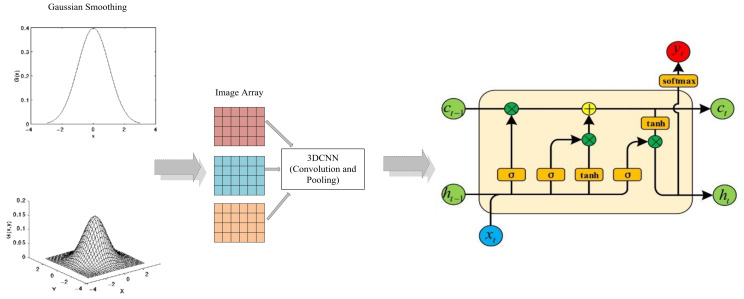
Architecture of the novel three-dimensional convolutional neural network combined with long and short-term memory network based on Gaussian kernel smoothing method (GSCNN-LSTM) model used for polar vortex intensity prediction. The leftmost two coordinate images represent one-dimensional Gaussian kernel smoothing (GS) and two-dimensional GS process, respectively. The middle part represents the input of image matrix and three-dimensional convolution neural network (3DCNN), and the rightmost represents the structure of long-term and short-term memory (LSTM) network.

Similar to many advanced DL neural network models, we apply one-dimensional GS and two-dimensional GS to the 3DCNN and LSTM network. By adjusting the method and sequence of network training, a more accurate method for extracting polar vortex image features and predicting polar vortex intensity index series is constructed. The construction of convolution neural network with long short-term memory based on Gaussian smoothing (GSCNN-LSTM) model is shown in Figure 1. Firstly, the polar vortex intensity index sequence is input into the model as a training set, which is a three-dimensional shape (training × 20 × 1); since our predicted time step is 20, we need to output the polar vortex intensity index series for 20 days. The polar vortex image is directly input into 3DCNN after two-dimensional GS, and the distribution characteristics of polar vortex potential height anomaly are extracted by convolution neural network. Finally, LSTM is used to predict multi-step time series.

**Figure 2:**
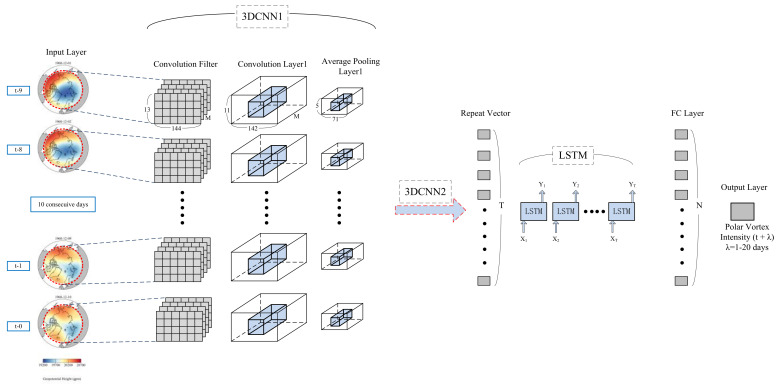
Prediction process of polar vortex intensity based on GSCNN-LSTM model. The GSCNN-LSTM model is composed of one-dimensional/two-dimensional GS preprocessing step in order to remove the noise of intensity time series and image data, two 3DCNN layers, two average pooling (AP) layers, a repetition vector (RV) layer, an LSTM layer and a full connection (FC) layer. The input variable is the daily geopotential height (GPH) average image data (in units of gpm) from day t to day t-9 (ten days in total), and the input GPH image range is 0–360° E and 60–90° N. Matrixes of 13 × 144 represent the length and width of the input image, respectively. The daily average polar vortex intensity from t + 1 to t + 20 (twenty days in total) is used as a variable for the output layer, and the blue three-dimensional structure in 3DCNN1 highlights the process of convolution. M denotes the number of feature maps, T represents the number of neurons in the RV layer, and N represents the number of neurons in the FC layer. Noted that N was set to 20 or 40 in this study.

In order to further explain the details and training process of the neural network, we show the setting of various parameters in the novel model, the convolution process of polar vortex image, and the prediction process of polar vortex intensity index in the training process of the neural network model. As shown in Figure 2, the specific prediction process of GSCNN-LSTM model is as follows: Firstly, take the polar vortex distribution image after GS as the input datasets of the model, and select the polar vortex image of the first ten days, that is, the polar vortex image from day t to day t-9. Since the polar latitude range selected in the research process is 60–90° N and the longitude range is 0–360° E, the image matrix is expressed as a matrix form of 13 × 144 according to the resolution of NCEP reanalysis data. When we divide the size of training set and test set data, we input the data from 1948 to 1997 as the training set, the data from 1998–2007 as the validation set, and the data of the last 13 years from 2008–2020 as the test set. Since the input time step is 10 and the predicted time step is 20, the number of samples per year will be reduced by 30, and the number of samples in the processed training set is 92 × 15 + 91 × 45 = 5475. The input layer shape is expressed as ten consecutive days of 5475 × 10 × 144 × 13 × 20. Then, we put each image through the set 3DCNN1 convolution kernel (set to 40 in this study), which represents the filter in 3DCNN1. After the operation of the convolution kernel, we need to merge all feature maps. However, in order to more clearly show the structure and calculation process of the neural network, we display the processing methods of each time step separately. The shape size is 5475 × 10 × 144 × 13 × 20 maps. Then, the convolution operation is carried out by using the convolution layer. In this study, the size of the convolution kernel is set to 3 × 3 × 3; after the first convolution layer, further extraction of the features of the output 3D structure and reduction of the amount of calculation is necessary, so we carry out average pooling processing, and obtain the data with shape of (None, 4, 71, 5, 40). None here represents the size of the number of samples. Due to the samples needing to be input into the model for training by batch processing during the training process, the None is not shown here, and the batch size is set to 40 in this study. Note that all trainable parameters in the 3DCNN model are initialized randomly, and then trained by online back propagation (BP) algorithm. After 3DCNN1 outputs the feature map, the same convolution operation is adopted to input the output shape to the 3DCNN2 layer. The obtained feature maps are (None, 20, 680) after repeating the repeat vector layer. Then, in order to extract the timing features, the three-dimensional data is further input into the LSTM layer. Here, the number of neurons in the LSTM layer is set to 80, which also represents the number of hidden layers. Ultimately, the intensity index sequence data of training output is obtained through a FC layer, and the number of neurons is set to 40. Twenty-day prediction time series of polar vortex intensity index indicates that the shape of output layer is (5475, 20, 1). In the testing program, the shape of the output layer used to predict is (1187, 20, 1).

The detailed neural network hyperparameters of the DL models in the training process are set as follows: The optimizer used in each neural network model in this experiment is Adam, the learning rate is 0.01 by default, the batch size is 40, and the number of epochs is 20. The early-stopping strategy is applied during the model training by obtaining the loss results on the validation set. With the increase of training epochs, if the loss in validation set does not improve in three epochs, the model will stop the training process. Thus, the “patience” hyperparameter value set for the early-stopping is three. As a result, less than 20 iterative experiments were stopped during the process of repeated experiments. In the last FC layer, L2 regularization and dropout method are used to prevent overfitting. It should be noted that the validation set is segmented from the training set in this study. The period of validation set is ten years from 1998 to 2007.

### 2.5. Model Comparison

In order to further distinguish and analyze the results of each model, we compare the differences between different DL models. The structure of the ConvLSTM model is quite different from the 3DCNN + LSTM model. Convlstm belongs to a variant of LSTM. It can be seen from the structure of LSTM in Equations (7)–(12), which include the variant of $x_{t}$ and $h_{t-1}$, and the FC layer inside the LSTM is directly used for input and output. ConvLSTM replaces the FC layer in LSTM with convolution calculation, which means the matrix multiplication is replaced by convolution calculation. The ConvLSTM models will capture the basic spatial features by convolution in multidimensional data in this way. The main difference between ConvLSTM and LSTM is the input dimension. Because the LSTM input data is one-dimensional, it is not suitable for spatial sequence data, such as video, satellite, and radar image datasets. ConvLSTM is designed for 3D data as its input. When 3DCNN + LSTM deals with three-dimensional datasets, it will use the 3DCNN part of the model to extract the spatial characteristics of the input data (polar vortex images) at first, and then inputs the one-dimensional results from the 3DCNN model into the LSTM model for intensity prediction. The main difference between 3DCNN + LSTM and ConvLSTM is that the former only performs convolution calculation for input variant ($x_{t}$), while the latter does not process the convolution calculation for $h_{t}$.

The model structure of GSCNN-LSTM is similar to that of 3DCNN + LSTM, except that the data is preprocessed for denoising before input into the model. In this process, we improve the model to adapt to the multi-day input and multi-day output structure for polar vortex multi-day intensity prediction. It should be noted that GS processing can denoise the image efficiently; even though the convolution layer in 3DCNN can preprocess the input images, the preprocessing of time series needs one-dimensional GS for preprocessing before inputting the convolution results of 3DCNN into the LSTM model. In order to make the method simple, save the time of model training, and improve the training efficiency, it is feasible to preprocess the time series and images of polar vortex based on the model. GS preprocessing steps are added into the CNN and ConvLSTM model to provide a fair comparison with the proposed GSCNN-LSTM model.

### 2.6. Evaluation of Multi-Models

This study adopts two evaluation indexes widely used in the evaluation of DL model: Pearson correlation coefficient and mean absolute error (MAE). Pearson correlation coefficient, also known as Pearson product moment correlation coefficient, is a linear correlation coefficient and the most commonly used correlation coefficient. Ham et al. [30] and others use Pearson correlation coefficient to express the correlation between the predicted El Niño 3.4 index and the real index, so as to further verify the effect of model prediction. It is used to reflect the linear correlation degree of two variables, X and Y, and the value of the Pearson correlation coefficient is [−1,1]. The greater the absolute value is, the stronger the correlation is.

(18) $$\rho_{X,Y}=\frac{cov\left(X,Y\right)}{\sigma_{X}\sigma_{Y}}=\frac{E\left[\left(X-\mu_{X}\right)\left(Y-\mu_{Y}\right)\right]}{\sigma_{X}\sigma_{Y}}$$

The value of Pearson correlation coefficient is expressed by Equation (18); $cov\left(X,Y\right)$ is the covariance of two variables, and the denominator is the product of the standard deviation of two variables. $\mu_{X}$ represents the average of X, $\mu_{Y}$ represents the average of Y, and $E\left[\left(X-\mu_{X}\right)\left(Y-\mu_{Y}\right)\right]$ represents the expectation.

MAE refers to the average of the absolute value of the error between the predicted value and the real value. Its calculation equation is as follows:

(19) $$MAE=\frac{1}{N}\overset{N}{\underset{j=0}{\sum }}\left|{\hat{y}}_{j}-y_{j}\right|$$

The smaller the error between the predicted value of the model and the real value is, and the smaller the MAE value is, the better the effect that the model prediction will achieve. In this study, it means that a more accurate polar vortex intensity index sequence is predicted by GSCNN-LSTM model.

In this study, we also apply the two model evaluation indexes to 3DCNN, ConvLSTM, 3DCNN-LSTM, and GSCNN-LSTM models to evaluate the error of prediction results of each model and make comparison. In the training process, we also repeated the training, by adjusting the parameters of each model, and recorded the Pearson correlation coefficient and MAE in the training process to evaluate the stability of each model.

## 3. Results

### 3.1. Segmentation of Datasets

**Figure 3:**
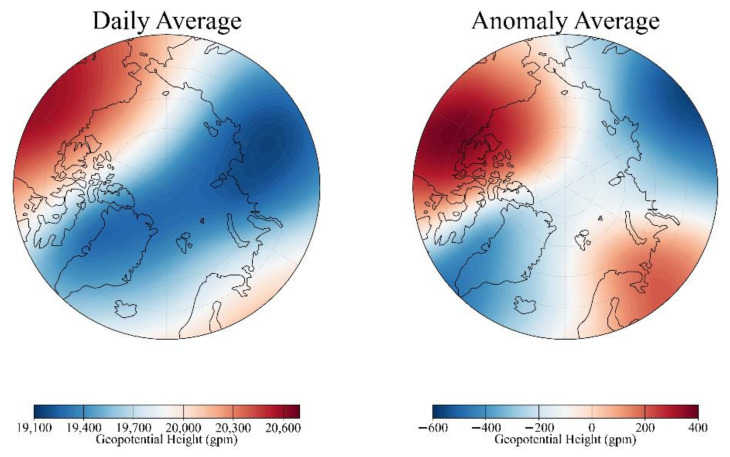
Horizontal distribution of daily-averaged (left panel) and daily anomaly-averaged (right panel) GPH of polar vortex of 1 February of 1948 at 50 hPa from NCEP reanalysis.

The distribution of the polar vortex in the stratosphere presents the shape of a minimum region of GPH. As shown in Figure 3, the average GPH distribution of polar vortex on 1 February of 1948 illustrates an extreme domain at lower latitudes. Due to the influence of the dynamic field, surface temperature, and various weather systems in the atmosphere, the central position of the polar vortex presents a continuous migration process. At the same time, intensity is also changing periodically [1,19,24]. It can be seen from Equation (1) that the calculation of polar vortex intensity in this study uses the daily anomaly-averaged GPH relative to the interannual generation, which is closely related to the latitude of the grid point. The abnormal distribution of the polar vortex in Figure 3 shows that the polar vortex on that day is stronger than the annual average polar vortex. In the center of the polar vortex, the GPH is abnormally large. Because there are great differences in the intensity of polar vortex between different years, and the intensity of polar vortex is obtained from the abnormal distribution of GPH, we extract the characteristics of GPH abnormal distribution at first.

**Figure 4:**
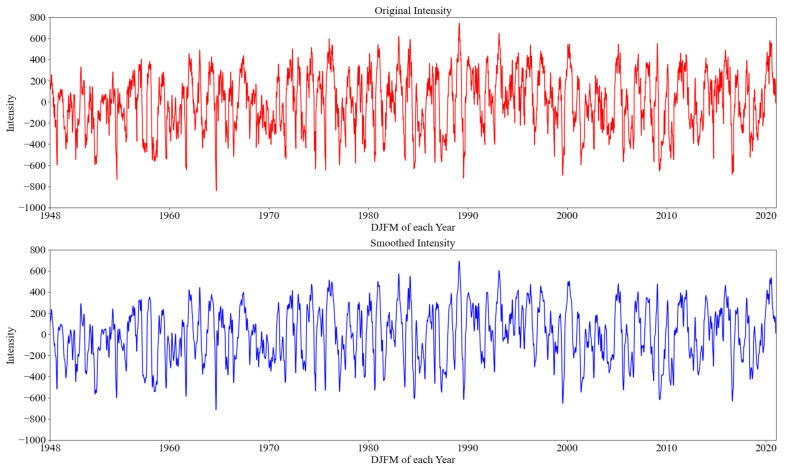
Time series of daily original intensity index (red line of upper panel) and the Gaussian kernel smoothed intensity index of polar vortex (blue line of lower panel) during January, February, March, and December (DJFM) for 1948–2020 from NCEP reanalysis datasets.

**Figure 5:**
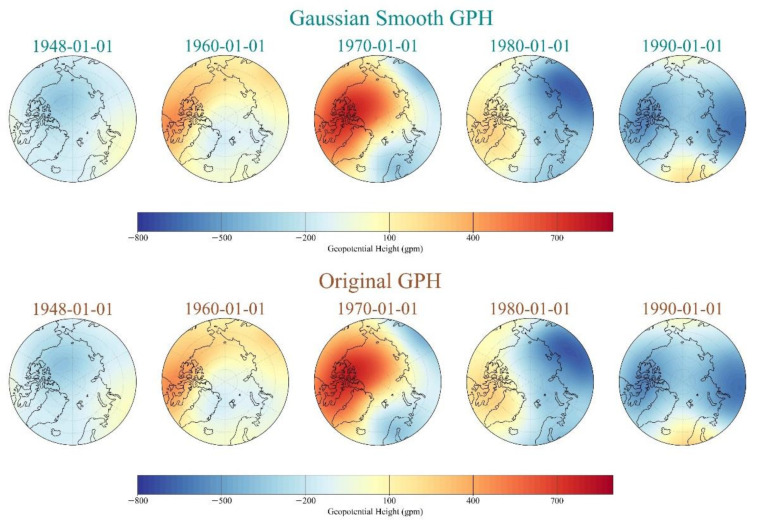
Horizontal distributions of original daily-anomaly-averaged GPH and Gaussian kernel smoothed daily-anomaly-averaged GPH of polar vortex at 50 hPa from 1948–1990. Each panel from left to right in both upper and lower row represents a distinct day of the polar vortex: 1 January of 1948, 1960, 1970, 1980, and 1990.

Because the sequence of polar vortex intensity index needs to be processed and divided, the one-dimensional Gaussian smoothing (GS) method is utilized for index time series denoising in this study. According to the calculation method of intensity index in 2.1, we constructed the standardized polar vortex intensity time series database by processing the DJFM data from 1948 to 2020 with the abnormal distribution of polar vortex GPH on the 100 hPa isobaric surface. As shown in Figure 4, the red and blue time series lines represent the variation of the original intensity index and the intensity index with Gaussian kernel smoothing, respectively. It can be seen that there is an obvious seasonal variation in the intensity of the polar vortex, which illustrates a trend of increasing first and then decreasing. However, from the perspective of interannual variation, there is no obvious interannual variation trend of polar vortex intensity, which also eliminates the influence of interannual variation in the process of training. We calculated that the standard deviations of the original data and the smoothed data were 265.92 and 254.70, respectively (not shown). It demonstrates that the dispersion of smooth data is reduced to a certain extent, and the effect of denoising is achieved. After denoising the time series and the prediction variables of DL, we also need to carry out two-dimensional GS to smooth the image data of polar vortex anomaly GPH. Figure 5 shows the distribution of some original polar vortex anomaly GPH and the distribution of two-dimensional Gaussian smoothed polar vortex anomaly GPH after the corresponding date. After constructing all polar vortex anomaly distribution maps, we generate an image database, which is divided with the corresponding intensity time series as the input of the new model.

As shown in Table 1, image data and intensity time series data are divided into two parts: training set and test set. Since the selection of months each year is not continuous, 121 days of data are selected in normal years and 122 days in leap years. The training period is 60 years, from 1948 to 2017, while the test set is 13 years, from 2008 to 2020. Since this study is based on multi-day to multi-day prediction, by subtracting the length of 10-day series input and 20-day prediction series output every year, the total number in the training sample sets is 5475 days and the total number of test sample sets is 1187 days. The maximum, minimum, and average values of the two time series samples are shown in Table 2. Compared with the magnitude of intensity, the average intensities of the two types of samples are −1.47 and 6.97, respectively. Both of them are close to zero, which is reasonable for each training model.

In order to speed up the convergence rate of the DL algorithm, the data are often normalized before training to speed up the solution rate of gradient descent. For the time series data of polar vortex intensity, we scale each value into [0,1]. The specific calculation equation is as follows:

(20) $$x^{\prime }=\frac{x-min\left(x\right)}{max\left(x\right)-min\left(x\right)}$$

For the image processing of polar vortex anomaly distribution, the same method is applied. Furthermore, the authenticity of the normalized image data is preserved to the greatest extent.

### 3.2. Model Training and Analysis

#### 3.2.1. Model Comparison

After dividing the training set and test set and smoothing the original data, we need to reshape the reconstructed polar vortex intensity series and image database before inputting the images and time series data into the model. The input shape is divided into time steps and predicted. Therefore, according to the model training method in Section 2.4, we predict the polar vortex intensity index for multi-day through the polar vortex GPH anomaly distribution in the first ten days. From the results of several model tests, the forecast lead time can be up to twenty days. Note that, in order to increase the total amount of training samples as much as possible when creating sequence data and image data, we set the sliding window to one step. Thus, even in the 122/121 days sequence every year, 91/92 samples can be generated. For verifying the accuracy and stability of the prediction results of the new model, this study compares the correlation results of traditional DL models with GSCNN-LSTM. In this study, the 3DCNN model is used for training primarily. After determining the size of the convolution kernel and average pooling layer parameters, we adjust each parameter, such as learning rate and epochs, to the most appropriate value that is suitable for polar vortex intensity prediction, and finally obtain the best CNN prediction results. Similarly, for the more advanced DL model ConvLSTM, we also adopt the same parameter adjustment method. Finally, the GSCNN-LSTM proposed by this research was used for training and predicting. The results show that the GSCNN-LSTM model we used has better prediction results than the traditional DL spatiotemporal training model and some improved models.

**Figure 6:**
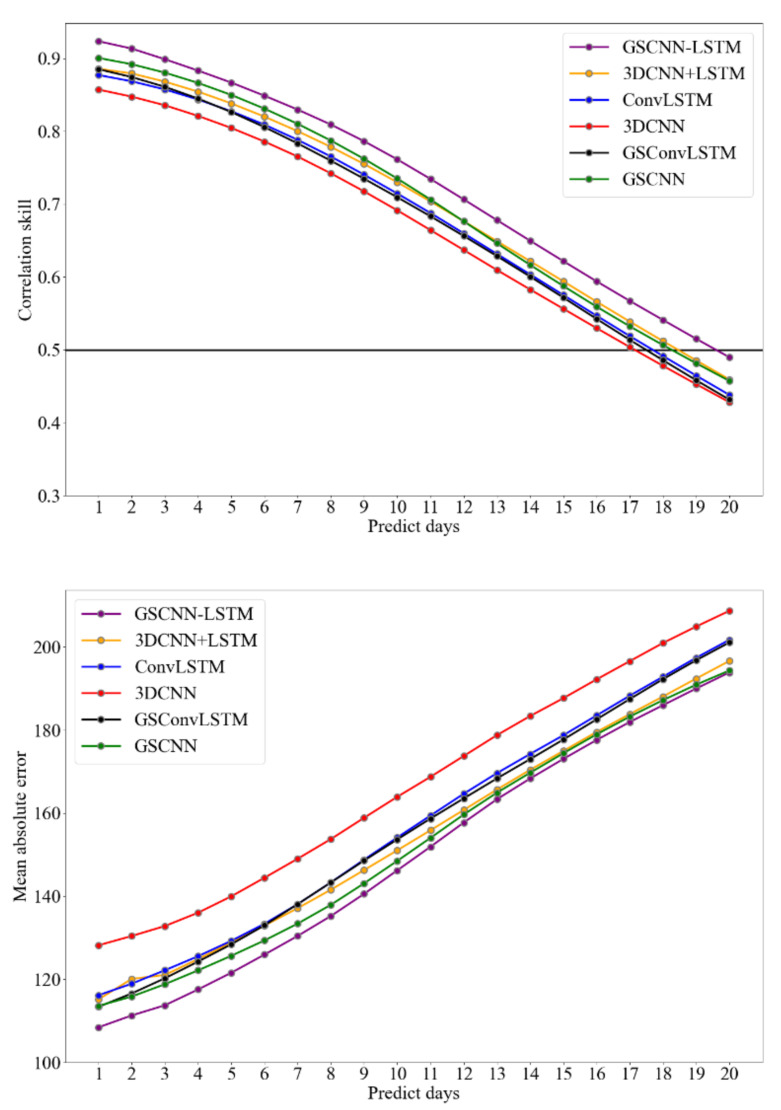
Polar vertex intensity over DJFM from 2008–2020 correlation skill in different DL models. The upper panel represents the correlation skill of the ten-day moving-averaged polar intensity index as a function of the forecast lead day in the 3DCNN model (red), convolutional LSTM (ConvLSTM) model (blue), GSConvLSTM (black), 3DCNN + LSTM model (yellow), GSCNN (green), and GSCNN-LSTM model (purple). The lower panel represents the mean absolute error (MAE) variation between real intensity and predicted intensity with the forecast lead days. The test period is between 2008 and 2020. Note that the forecast time has reached 20 days.

Figure 6 shows the correlation and mean absolute error (MAE) results of polar vortex forecast lead 20-day obtained from 3DCNN, GSConvLSTM, ConvLSTM, 3DCNN + LSTM, GSCNN, and GSCNN-LSTM models. The results show that the prediction effect decreases with the prolonging of predicting time in all training models. From the data where the correlation gradually decreases with the prediction duration, the prediction intensity time series obtained by the GSCNN-LSTM model proposed in this study is more consistent with the original data, and always maintains a high level of correlation with the forecast lead time on the same day. Note that all correlation coefficients in this paper are Pearson correlation coefficient, which is calculated by Equation (18). The predicted polar vortex intensity of a one-day lead can be highly correlated with the original sequence which can up to 0.92. With the increase of forecast lead days, only the GSCNN-LSTM model can reach the correlation coefficient close to 0.5 when the polar vortex intensity forecast lead time is up to 20 days. In order to design a fair comparison in different DL models, all traditional and advanced DL models used a denoised version of the input image data and predicted a denoised polar vortex intensity. Thus, GS preprocessing steps were also added into the CNN model and ConvLSTM model, which illustrated a relatively lower accuracy than the correlation skill of the GSCNN-LSTM model. The MAE series plot can also derive the same conclusion, which means the prediction error of GSCNN-LSTM model is still better than other DL models with the increase of forecast lead time.

#### 3.2.2. Ablation Experiment

In order to prove that the GSCNN-LSTM model proposed in this paper possesses a preferable and reliable result for the prediction of polar vortex intensity, ablation experiments are carried out. This study adopts the method of ablation experiment to show the importance and necessity of each component (GS preprocessing, 3D-CNN, and LSTM) in the proposed model, and a comparative test is proposed by adding a simple five-point smoothing (FS) during the preprocessing phase of 3DCNN + LSTM model for highlighting the importance of GS preprocessing of the GSCNN-LSTM model.

**Figure 7:**
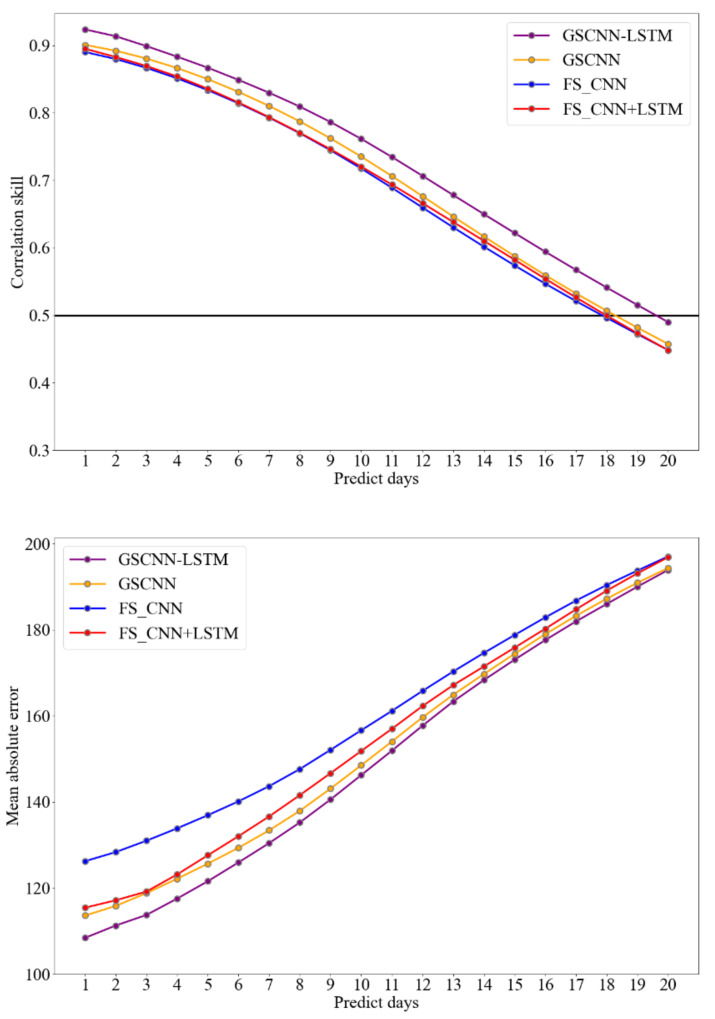
The same as Figure 6, but for different training models. In both upper and lower panels, the purple line represents the result of the GSCNN-LSTM model, the yellow line represents the result of the GSCNN model, the blue line represents the result of five-point smoothing with CNN (FS_CNN) model, and the red line represents the result of five-point smoothing with CNN + LSTM (FS_CNN + LSTM) model.

After a simple FS preprocessing is added to the 3DCNN and 3DCNN + LSTM model, it is necessary to compare with the experimental results obtained by one-dimensional and two-dimensional GS denoising process primarily. Furthermore, in order to highlight the importance of LSTM for long time series prediction in the process of polar vortex intensity index prediction, FS combined with CNN model (FS_CNN), FS combined with CNN + LSTM (FS_CNN + LSTM), and GSCNN model are also proposed for a comparative test. The results of the ablation experiment are shown in Figure 7. The results show that each component of the GSCNN-LSTM model is indispensable. Integrating GS into the DL methods can obtain a more accurate prediction result than simple smoothing (such as FS), and by comparing the prediction results of the FS_CNN + LSTM and FS_CNN model, we can draw the conclusion that LSTM has a good performance for polar vortex intensity prediction, and it is also an important component for the GSCNN-LSTM model to accurately capture the characteristics of intensity index time series. The same conclusion can be reached from the prediction results of CNN, GSCNN, and GSCNN-LSTM models.

**Figure 8:**
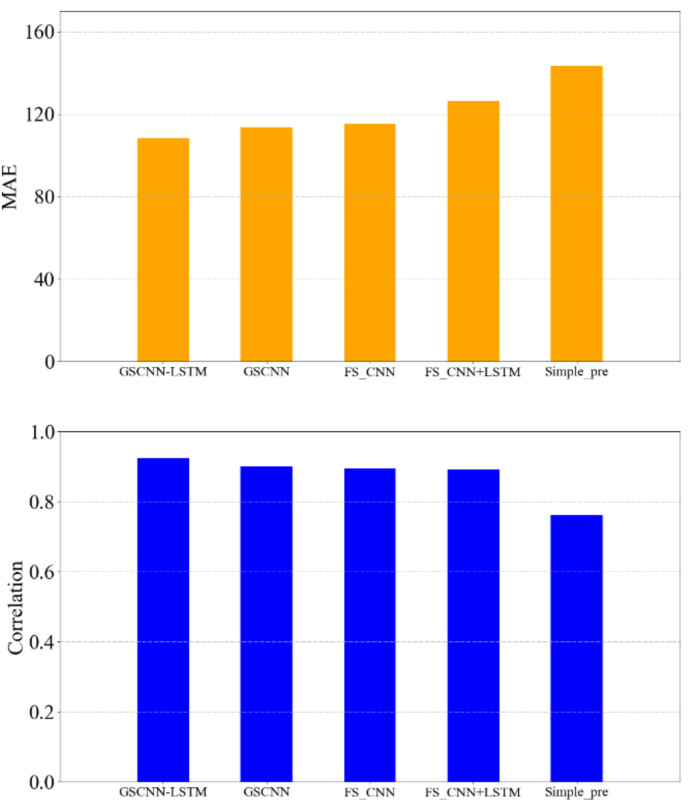
The upper panel is the MAE corresponding to each model experiment result, and the lower panel is the Pearson correlation coefficient.

After completing the ablation experiment, we also added a simple basic prediction model to compare with other DL models. The average value of the last twenty days’ polar vortex intensity is taken as the predicted intensity. In the different DL models, we choose the time series of the last twenty days as the precondition for prediction. Therefore, it is necessary for the simple experiment to set a twenty-day precondition for a fair comparison with DL models. As shown in Figure 8, the prediction result of the simple model (Simple_pre) is relatively poorer than the DL models, its Pearson correlation coefficient is relatively low, and MAE relatively high. Therefore, DL models and the proposed GSCNN-LSTM model are feasible to predict the polar vortex intensity.

### 3.3. Model Validation

After evaluating the accuracy of model training, we need to evaluate the stability of the GSCNN-LSTM model. In the process of training the model, it is often necessary to find a suitable neural network to extract spatial features more effectively. Besides, the more training parameters the model possesses, the greater the amount of the calculation is. However, the accuracy may not be effectively improved. Table 3 shows the results of each model in the training process, which provides a basis for selecting the optimal model to predict the polar vortex intensity index. Due to the limitation of time step and the increase of network layers, we choose (3 × 3 × 3) as the convolution filter. The convolution kernel is used as the convolution layer parameter of all 3D structure models. The results show that applying fewer LSTM hidden units can reduce the training parameters and shorten the training time, and the best prediction effect can be achieved in the proposed new network GSCNN-LSTM. Even under different parameters, the GSCNN-LSTM model is better than other models in long-term training prediction. The correlations of one day, five days, and twenty days in advance can reach more than 0.9, 0.85, and 0.48 respectively, which are higher than those of some ensemble models such as 3DCNN + LSTM and ConvLSTM.

**Figure 9:**
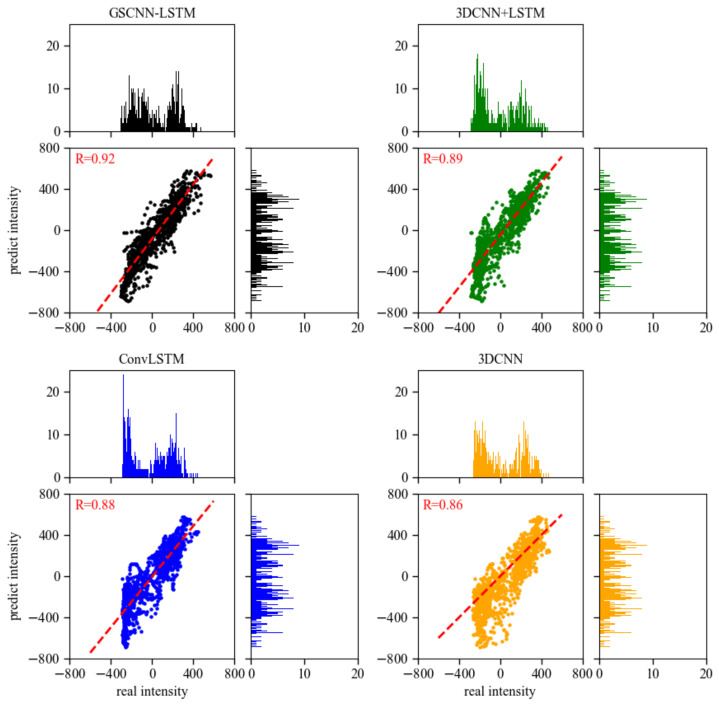
The scatter–histogram plot of the real polar vertex intensity and the forecast lead one-day intensity results from GSCNN-LSTM, 3DCNN + LSTM, ConvLSTM, and 3DCNN models in DJFM of 2008–2020. Black dots represent the GSCNN-LSTM model, green dots represent the GSCNN-LSTM model, blue dots represent the GSCNN-LSTM model, and orange dots represent the GSCNN-LSTM model. The histogram represents the number of times the polar vortex intensity data overlap in each interval of 3. The dashed red line shows a linear fit. Correlations for the linear fit are given in the upper left corners of the panels.

From the optimal model, the correlation with the highest predicted one-day intensity index of 0.92 is obtained, and then the correlation is analyzed using the scatter–histogram plot. As shown in Figure 9, the position of the scatter points represents the concentration degree between the predicted intensity index results and the true value. The histogram counts the number of scatter points with an interval of three. The results show that, compared with the other three models, the intensity index predicted by the GSCNN-LSTM model is closer to the real intensity value, which can be evenly distributed in the interval and has a higher concentration degree with the real intensity. The four models all show unsatisfactory prediction results in low-value intensity areas, but in other intensity areas, the predicted intensity of the GSCNN-LSTM model can be well separated, and the distribution is relatively uniform, which means the model can better extract the characteristics of intensity time series and polar vortex images.

**Figure 10:**
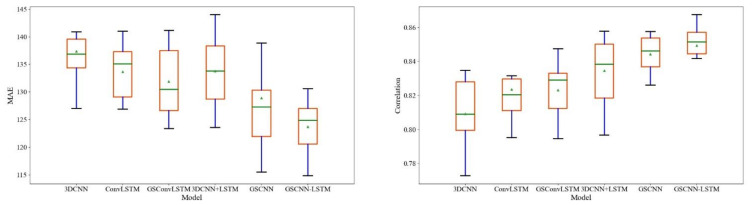
The boxplot of prediction results of mean absolute error (MAE) and correlation with different models.

After evaluating the intensity of the model prediction and the concentration degree with the real intensity, the model needs to be repeatedly trained to verify whether the optimal model has strong stability. Figure 10 shows the correlation and MAE box diagram of predicted polar vortex intensity generated by GSCNN-LSTM, GSCNN, 3DCNN + LSTM, GSConvLSTM, ConvLSTM, and 3DCNN models after repeated training. The upper edge, lower edge, median, 25% median, and 75% median of each group of MAE data are shown in Figure 10. The results show that by changing the training dataset and the verification dataset, the uncertainty of the prediction skills generated by the GSCNN-LSTM model is minimal, indicating that the GSCNN-LSTM model can provide proficient real-time predictions, and the model has high stability. The prediction results of the ConvLSTM, GSConvLSTM, and 3DCNN + LSTM model are relatively close, but the training efficiency of ConvLSTM and GSConvLSTM are relatively lower than that of GSCNN and GSCNN-LSTM, and the training time is too long, and overfitting often occurs due to the many training parameters of ConvLSTM and GSConvLSTM model. LSTM always illustrates a positive effect on the prediction of long-time intensity series by comparing the training results of GSCNN and GSCNN-LSTM. Therefore, the improved set model is also carried out on 3DCNN + LSTM.

## 4. Discussion

In this study, a new DL model algorithm for time series prediction is applied to the multi-day prediction of the polar vortex. In the long-term polar vortex sequence prediction, the GPH anomaly distribution features of the polar vortex can be better extracted by the GS algorithm and the training results of the improved 3DCNN-LSTM model, which provide a more accurate and stable result for multi-day prediction while reducing the image information and timing complexity. Compared with the traditional CNN model and its variant set model, this model shows excellent performance and emphasizes the combination of training data processing and model in the spatiotemporal information extraction.

(1) The increase/decrease of the polar vortex can also be attributed to the process of entropy increasing/decreasing. In this process, more attention is paid to the variation of the physical state of the polar vortex. Therefore, advanced insight is provided for the capture and prediction of the intensity of the polar vortex and weak/strong events. The strong ability of the neural network to learn characteristic law can be used to further predict the polar vortex intensity variation in the process of the changes that occur to polar vortex morphology and position, which can further explain the laws of its physical development and provide more atmospheric models with practical prediction significance. The stability and accuracy of the GSCNN-LSTM model can further show the application prospect of the ensemble model and provide a reference for the prediction of atmospheric eddy system with entropy increasing/decreasing.
(2) Based on the nonlinear physical characteristics of the atmospheric vortex systems and weather phenomena, the GSCNN-LSTM model can effectively remove the less influential factors in the physical features combined with the traditional mathematical method, and then use the 3DCNN network of multi-time step prediction to capture the abnormal distribution features of GPH and extract long-term impact factors from the LSTM. As a result, the prediction of atmospheric eddy systems has been significantly improved. This is also widely demonstrated in TC intensity, eddy identification, cloud detection, and synoptic-scale eddy studies, e.g., [58,64,80,93]; for example, the multiscale feature fusion method can achieve about 98% accuracy of ocean eddy detection [93]. The improved method of CloudLSTM’s novel recurrent neural network can also provide an accurate long-term prediction of air quality indicators [80]. The new DL method proposed by us also has a significant effect on capturing the characteristics of nonlinear systems in the atmosphere.
(3) Arctic sea ice loss is closely related to global atmospheric circulation and climate warming. Studies have shown that the polar vortex has a strong negative phase response to the loss of sea ice, and then influences the mid-latitude surface temperature through large-scale circulation such as AO [16]. These responses often lead to extreme weather events. The multi-day prediction results of the polar vortex intensity index provide a theoretical basis for the long-term climate change trend and numerical weather forecast results, and the correlation between the two will also provide a reference for the accurate quantification of global temperature change and extreme precipitation events.
(4) It is rare to apply the signal or image processing method in mathematics to the model for time series prediction. In this study, the prediction accuracy is applied to a network model through a GS method, which can not only remove the noise in the sequence, but also reduce the redundant information in the images. Compared with the traditional signal denoising method, the advanced Gaussian denoising method has the ability to process multidimensional data and can also be widely used in the fields of artificial intelligence and atmospheric science, as well as other scientific fields. Therefore, the GSCNN-LSTM model provides a good reference for improving the prediction method of vortex index by combining with DL methods.
(5) From the perspective of prediction results, we can improve the forecast lead time of polar vortex intensity to 20 days while ensuring prediction accuracy. The definition method of strong and weak polar vortex events is usually defined by extremely weak or very strong polar vortex for 20 consecutive days [1,23], and polar vortex events can reflect the variability of the polar vortex and have a periodic impact on chemicals, e.g., [13,23]. Therefore, in future research, we can consider adding the prediction of polar vortex intensity events to ensure the accurate prediction of intensity index, further improve the accuracy of predicted events, and simulate the important impact of polar vortex intensity variability in historical periods. It provides a feasible scheme for the study of atmospheric circulation in the NH.
(6) However, the paper also has some limitations, such as the lack of consideration of multiple predictors. It is not a feasible method to add El Niño index and AO index into the model due to the different latitudes of the characteristics. However, they also exert a significant influence on the variation of polar vortex intensity and position morphology. Therefore, the model with multiple predictors should be further discussed in future research. For example, the images of sea surface temperature (SST) anomalies, sea ice coverage, potential vorticity (PV), and wind field in the Arctic region are input into the model, and the most relevant variables are selected by advanced feature extraction methods. In this study, the prediction effect is relatively poor when the polar vortex intensity is negative exponential. These variables may improve the prediction of the negative intensity index to some extent.
(7) Furthermore, the GSCNN-LSTM model needs to be improved. The optimization based on the GSCNN-LSTM model needs to learn from the idea of the aggregate model, which can effectively improve the prediction effect and shorten the training time with the addition of more variables, namely prediction factors, so as to make a timely response to the weather system changes. Some recent research and improved methods can be used as references for future research on vortices, such as the benchmark model for short-time precipitation forecasting proposed by [94]. The prediction of polar vortex intensity can be improved by using the method of the benchmark model. The combination of the self-attention mechanism and the ConvLSTM model adopted in spatiotemporal prediction achieves state-of-the-art results [95]. Using the most advanced attention mechanism to predict a certain day or a certain strong/weak event of the polar vortex in a long time series may achieve better results. This method of model fusion may be widely used in future research and can create new achievements continuously, providing strong support for numerical weather prediction.
(8) Ultimately, the polar vortex is an important path for atmospheric dynamic transmission and substance exchange in the chaotic system of the atmosphere. This paper provides a significant theoretical basis for the nonlinear dynamics and entropy increase theory in the atmosphere through revealing the intensity variation of the polar vortex. According to the results of the GSCNN-LSTM model, the intensity information and variation characteristics of the polar vortex were effectively extracted and predicted, which proves that the energy information of many vortex systems of atmosphere can be predicted by DL models. For example, Liu et al. [96] and others used the CNN + LSTM method to extract the temporal and spatial features of partial discharge input signal, which improved the accuracy of partial discharge signal pattern recognition. The accurate numerical prediction is inseparable from the research of information entropy theory in the atmosphere. Combined with the research method of DL, many prediction problems in atmospheric science can be further solved.

## 5. Conclusions

In this study, a new DL model is proposed to predict the variation of multi-day intensity of polar vortex in a more accurate and stable way. Firstly, the dataset of polar vortex intensity and image distribution are constructed by using the long-term NCEP/NCAR historical reanalysis data. Then, the images and intensity series are divided and input into a three-dimensional convolutional neural network combined with long and short-term memory network based on Gaussian smoothing method (GSCNN-LSTM) model for training and prediction. According to the idea of ensemble models and the construction method of the advanced model, and fully considering the temporal and spatial distribution characteristics of the polar vortex, a high-quality and high-precision DL method for polar vortex intensity index prediction is obtained. During the training process, we input the one-dimensional intensity index time series data disposed by GS and polar vortex GPH anomaly distribution images in the model, extract the features through two-layer 3DCNN convolution and pooling layer, then the repeated vector layer of the data inputs the shaped data into LSTM to extract the time series features, and finally outputs the multi-day polar vortex intensity prediction results. Compared with some traditional and advanced DL methods, the novel GSCNN-LSTM model can obtain a high correlation between the predicted sequence and the original sequence and more accurate prediction results, according to the Pearson correlation coefficient and MAE results. Secondly, the forecast lead time of the 20-day intensity series is the limitation, which can achieve a correlation of 0.49. Finally, the model also illustrates a good performance in stability, and it is better than the general DL algorithm models such as 3DCNN and ConvLSTM. The novel algorithm model can provide some guidance for the prediction of many nonlinear systems in the atmosphere.

However, this article still has many limitations. As revealed by the previous studies, many impact factors are often considered in the prediction process of diverse vortex systems, such as the temperature of the underlying surface, the dynamic field, and the interaction between different systems [61,62,63,64]. In this paper, many weather systems affecting the intensity of polar vortex are not considered in detail. Therefore, referring to the prediction of TC intensity and track, multifactor prediction for polar vortex needs to be explored and improved. Secondly, the recent proposed DL network is not used to extract the polar vortex image characteristic. Even if the correlation of the training results has achieved good results, the value of predicted polar vortex intensity still needs improvement, and the advanced image neural network needs to be further explored.

According to the hypothesis proposed in this paper, although vortex systems in weather systems have similar dynamic structures and characteristics with polar vortex, the impact factors are different, so the generalization ability has not been well verified at first. Further works are needed to explore more innovative DL models and improve their generalization ability. Secondly, according to the hypothesis of improving DL models, this paper proposes a GS method combined with the DL model, which has achieved good results in polar vortex prediction. Finally, for the hypothesis of adding appropriate variables to the model, the addition of temperature field, potential vorticity, and dynamic field cannot improve the prediction effect of the model and can even reduce the accuracy of the DL models, so the appropriate prediction factors of polar vortex intensity need to be further studied.

In this paper, the polar vortex is taken as the research object, which is the most representative vortex system in the Northern Hemisphere (NH). In future research, we can apply this method and diverse improved models to forecast many small and medium-size vortex systems, including the intensity prediction of TC and extratropical cyclones. DL models are commonly used in the image prediction field [97,98], which is similar to the prediction of ENSO events [30]. The intraseasonal scale prediction of polar vortex intensity in this paper can be further developed into the prediction of strong/weak polar vortex events.

## Figures and Tables

**Table 1:** The CNN and LSTM notable applications.

Reference	Methods	Application
Paritosh Parmar et al., 2021 [68]	2D-CNN	Hallucinate spatiotemporal representations
Kong, Z. et al., 2020 [69]	CNN	Condition monitoring of wind turbines
Fanhua Yu et al., 2021 [70]	3D-CNN	Spatiotemporal soil temperature forecasting
Ajami, A. et al., 2019 [71]	CNN	Identifying a slums’ degree of deprivation from VHR images
Jose Selvi et al., 2021 [72]	LSTM	Detecting algorithmically generated domain names
Isaiah Oyewole et al., 2021 [73]	Hybrid LSTM	State-of-charge estimation of Li-ion batteries
Ghimire, S. et al., 2019 [74]	LSTM	Deep solar radiation forecasting
Zhang, R., et al., 2019 [75]	LSTM	Nonlinear structural seismic response prediction

**Table 2:** Statistical parameter of training and testing datasets of polar vortex intensity, including period and number of each divided datasets (note that, due to the select number of timesteps being ten, and the prediction lead time being twenty days, the daily number of JJA is reduced to 122/121 minus 30, which is equal to 92/91). The maximum, minimum, and mean intensity values of each dataset are shown.

Data (After Dispose)	Number (Year × Days)	Period
Training	92 × 15 + 91 × 45 = 5475	1948–2007
Testing	92 × 4 + 91 × 9 = 1187	2008–2020
**Data (After Dispose)**	**Max Intensity–Min Intensity**	**Mean Intensity**
Training	745.41–841.76	−1.47
Testing	579.15–688.38	6.79

**Table 3:** Comparison of 3DCNN, ConvLSTM, 3DCNN + LSTM, and GSCNN-LSTM models on the polar intensity dataset. “−3 × 3 ×3” and “−5 × 5” is consistent with “3 × 3 × 3” and “5 × 5”, which represent the corresponding kernel size of the neural network layers. “L120”, “L100”, and “L80” refer to the number of hidden state in the LSTM layers, and “40” and “20” refer to the number of output filters in the convolution.

Model	Number of Parameters	Correlation of Forecasting Lead 1-Day	Correlation of Forecasting Lead 5-Day	Correlation of Forecasting Lead 20-Day
3DCNN(3 × 3 × 3) 3 × 3 × 3-40-3 × 3 × 3-20	29,561	0.85	0.80	0.42
ConvLSTM(5 × 5) 5 × 5-40-5 × 5-20	556,281	0.87	0.82	0.43
ConvLSTM(3 × 3) 3 × 3-40-3 × 3-20	606,521	0.88	0.82	0.44
3DCNN + LSTM(3 × 3 × 3) 3 × 3 × 3-40-3 × 3 × 3-20-L120	412,101	0.88	0.83	0.46
3DCNN + LSTM(3 × 3 × 3) 3 × 3 × 3-40-3 × 3 × 3-20-L100	339,221	0.89	0.84	0.46
GSCNN-LSTM(3 × 3 × 3) 3 × 3 × 3-40-3 × 3 × 3-20-L120	412,101	0.91	0.86	0.48
GSCNN-LSTM(5 × 5 × 5) 3 × 3 × 3-40-3 × 3 × 3-20-L100	339,221	0.92	0.86	0.48
**GSCNN-LSTM(3 × 3 × 3)** **3 × 3 × 3-40-3 × 3 × 3-20-** L80	**269,541**	**0.92**	**0.87**	**0.49**

## Data Availability

The data which can support the study results is available in https://psl.noaa.gov/cgibin/db_search/DBListFiles.pl?did=198&tid=92182&vid=663, accessed on 10 August 2021.
